# Blockage of SLC31A1‐dependent copper absorption increases pancreatic cancer cell autophagy to resist cell death

**DOI:** 10.1111/cpr.12568

**Published:** 2019-01-31

**Authors:** Ze Yu, Rongtao Zhou, Yicheng Zhao, Yi Pan, Hao Liang, Jin‐San Zhang, Sheng Tai, Liang Jin, Chun‐Bo Teng

**Affiliations:** ^1^ College of Life Science Northeast Forestry University Harbin China; ^2^ State Key Laboratory of Natural Medicines Jiang su Key Laboratory of Drug Screening School of Life Science and Technology China Pharmaceutical University Nanjing China; ^3^ Department of Hepatopancreatobiliary Surgery Second Affiliated Hospital of Harbin Medical University Harbin China; ^4^ School of Pharmaceutical Sciences and the Center for Precision Medicine The 1st Affiliated Hospital Wenzhou Medical University Wenzhou China

**Keywords:** autophagy, copper, dormancy, pancreatic cancer, SLC31A1

## Abstract

**Objectives:**

Clinical observations have demonstrated that copper levels elevate in several cancer types, and copper deprivation is shown to inhibit tumour angiogenesis and growth in both animal models and preclinical trials. However, the content of copper in pancreatic duct adenocarcinoma (PDAC) and whether it is a potential therapy target is still unknown.

**Materials and Methods:**

The levels of copper in PDAC specimens were detected by ICP‐MS assays. Copper depletion in Panc‐1 or MiaPaCa‐2 cells was conducted via copper transporter 1 (SLC31A1) interference and copper chelator tetrathiomolybdate (TM) treatment. The effects of copper deprivation on cancer cells were evaluated by cell proliferation, migration, invasion, colony formation and cell apoptosis. The mechanism of copper deprivation‐caused cancer cell quiescence was resolved through mitochondrial dysfunction tests and autophagy studies. The tumour‐suppression experiments under the condition of copper block and/or autophagy inhibition were performed both in vitro and in xenografted mice.

**Results:**

SLC31A1‐dependent copper levels are correlated with the malignant degree of pancreatic cancer. Blocking copper absorption could inhibit pancreatic cancer progression but did not increase cell death. We found that copper deprivation increased mitochondrial ROS level and decreased ATP level, which rendered cancer cells in a dormant state. Strikingly, copper deprivation caused an increase in autophagy to resist death of pancreatic cancer cells. Simultaneous treatment with TM and autophagy inhibitor CQ increased cell death of cancer cells in vitro and retarded cancer growth in vivo.

**Conclusions:**

These findings reveal that copper deprivation‐caused cell dormancy and the increase in autophagy is a reason for the poor clinical outcome obtained from copper depletion therapies for cancers. Therefore, the combination of autophagy inhibition and copper depletion is potentially a novel strategy for the treatment of pancreatic cancer and other copper‐dependent malignant tumours.

## 1 INTRODUCTION

Copper is an essential trace metal element required for the activity of numerous biochemical enzymes in human cells. It is taken up mainly by the transmembrane copper transporter protein SLC31A1 (also known as CTR1), and then delivered by the copper chaperone for superoxide dismutase (CCS) to SOD1, which exerts an antioxidative role.^1^, ^2^ Moreover, intracellular copper ions could be transported by the antioxidant 1 chaperone protein (ATOX1) to the Golgi apparatus for excretion or be carried by the cytochrome c oxidase copper chaperone (Cox17) into the mitochondria to participate in cellular metabolism, energy generation and oxygen transport.^3^, ^4^

Abnormal accumulation of copper, however, is frequently observed in multiple cancers.^5^, ^6^, ^7^ A correlation has been established between increased levels of copper and the progression of several cancers.^8^, ^9^, ^10^, ^11^ In favouring tumour growth and angiogenesis, copper plays an integral role since it is revealed as the co‐factor for several pro‐angiogenic molecules like vascular endothelial growth factor (VEGF).^6^ In addition, copper ions also participate in oncogenic BRAF signalling in promoting tumour cell proliferation and migration.^12^

Intriguingly, copper‐specific chelators or inhibitors of ATOX1 and CCS have been demonstrated to be capable of suppressing the proliferation of several cancer cells.^9^, ^12^, ^13^ Tetrathiomolybdate (TM), a well‐tolerated copper chelator, is shown to inhibit tumour angiogenesis and growth in animal models and clinical trials.^14^, ^15^, ^16^, ^17^ Therefore, copper deprivation has been considered a hopeful strategy for the therapy of cancers with high copper content.

Pancreatic ductal adenocarcinoma (PDAC) is a highly malignant cancer, with a five‐year survival rate <5%.^18^ It has been reported that copper ions are increased in the blood specimens of patients with PDAC^10^, ^19^; however, the content of copper in PDAC and whether it is a potential therapy target are still unknown.

In this study, we report that copper and SLC31A1 coexist at a higher level in pancreatic cancer, and their levels were negatively correlated with the survival time of the patients. Blocking copper absorption via TM or knock‐down of SLC31A1 could inhibit pancreatic cancer progression by rendering them in a dormant state; however, cell death was not increased. Interestingly, we discovered that copper deprivation increased pancreatic cancer cell autophagy to resist cell death, which was likely a major reason for the unsatisfactory results in using copper deprivation strategies for clinical trials for cancers.

## 2 MATERIALS AND METHODS

### 2.1 Patients and clinical specimens

Human PDAC samples were obtained from sixteen patients after surgical resection at the Second Affiliated Hospital of Harbin Medical University in Harbin, China. Informed consent was obtained from the patients, and the research was approved by the appropriate committees of the Harbin Medical University.

Resected primary tumours were histologically examined by H&E staining. Histologically, all the tumours were invasive PDACs. None of the patients had received neoadjuvant chemotherapy. Fresh frozen samples were obtained for SLC31A1 expression analysis and immunohistochemistry analysis.

### 2.2 Cell lines and mouse treatment studies

The human pancreatic duct adenocarcinoma cell lines Panc‐1 and MiaPaCa‐2 were obtained from the American Type Culture Collection (Chicago, IL) and cultured in a complete growth medium. The cultured cells were maintained in a 5% CO_2_ atmosphere at 37°C. The Panc‐1 and MiaPaCa‐2 cell lines were authenticated by checking their gene expressions and were tested for the absence of mycoplasma contamination.

For the pancreatic cancer cell sphere culture, MiaPaCa‐2 cells in RPMI 1640 medium with 0.25% Matrigel were plated into a prepared 96‐well plate with 2% agarose in RPMI 1640 medium (5 cells per well) and cultured for 7‐14 days.

All animal experiments were conducted in accordance with the guidelines of the Animal Care and Ethics Committee of Northeast Forestry University. Male immune‐deficient (NPG) mice (4 weeks old) were received from the Beijing Vitalstar Biotechnology Co., Ltd. (Beijing, China) and fed in a sterile environment with food and water ad libitum. For xenografts, 2 million cells suspended in 100 μL of PBS were subcutaneously injected into the lower flank of the NPG mice. The cell‐transplanted mice received TM (500 μmol/L; 323446 Sigma, St. Louis, MO, USA) or chloroquine diphosphate salt (CQ; 100 μmol/L; C6628 Sigma) individual treatments, or TM (500 μmol/L) combined with CQ (100 μmol/L).

### 2.3 ICP‐MS assay

The measurement of elements was determined by a 7500 cx Agilent inductively coupled plasma mass spectrometry instrument (Agilent, Santa Clara, CA). All tissue or cell samples were dried for 48 hours at 60°C. Copper content assays were run in the reaction mode with high‐purity (>99.999%) ammonia as the reaction cell gas. Magnesium was used as the control element. A triple nickel cone interface and a quadrupole ion deflector emitted a tightly focused ion beam. The sample introduction system consisted of a quartz cyclonic spray chamber and a glass Type C nebulizer. The plasma torch argon purity (Linde Health‐care, Saint‐Priest) was higher than 99.999%.

### 2.4 Immunohistochemistry assays

IHC assays were performed on 8‐μm sections according to standard protocols.^20^ The SLC31A1 antibody concentration was 1:200 (sc‐66847, Santa Cruz, CA, USA). The photos were taken using a microscope at 200× and 400× magnification.

### 2.5 Bioinformatics analyses

The expression levels of copper transporters in pancreatic cancer and normal tissues were obtained via the MERAV database (http://merav.wi.mit.edu). The patient survival curve data were downloaded from the OncoLnc TCGA database (http://www.oncolnc.org/). The gene expression data regarding TM‐treated breast cancer cells (GSE77515) were downloaded from the GEO database. KEGG pathway analysis was performed through the online Database (http://www.genome.jp/kegg/pathway.html). Biochemical process was analyzed using the software FunRich.V3.

### 2.6 Real‐time RT‐PCR

Total RNA was extracted from the tumour samples and cells using the RNA reagent Trizol (Thermo Fisher Scientific, Waltham, MA, USA). Real‐time RT‐PCR was used with SYBR^®^ Premix Taq (Roche, Branchburg, NJ, USA) on a Roche 480 Real‐time PCR System. *β*‐actin was used as the endogenous control. The relative gene expression levels were quantified by normalization to endogenous *β*‐actin expression levels, which were calculated by the 2^‐ΔΔC(*t*)^ method.

### 2.7 Cell proliferation assay

Panc‐1 and MiaPaCa‐2 cells were transfected with NC or si‐Slc31a1 (50 nmol/L) or treated with TM (50 μmol/L) or CQ (10 μmol/L) in 96‐well plates. The viability of cells was evaluated by the CCK‐8 assay. The optical density of each well at 450 nm (OD450) was measured with a Microplate Reader (sunrise TECAN, JAPAN).

### 2.8 Wound healing assay

For the monolayer wound healing assays, the cells were plated in 24‐well tissue culture plates after transfection. Confluent monolayers were scratched using a small pipette tip and washed once with serum‐free medium. After 24 hours, migration was assessed microscopically.

### 2.9 Cell cycle analysis by flow cytometry

Panc‐1 and MiaPaCa‐2 cells were digested and collected 48 hours post‐transfection and washed with PBS twice. After being fixed in 70% ethanol at 4°C for 1 hour, the cells were resuspended in a staining solution of 50 μg/mL propidium iodide (PI), (Beyotime, Shanghai, China), 1 mg/mL RNase A, and 0.1% Triton X‐100 in PBS for 1 hour. The stained cells (1 × 10^5^) were then analyzed with a flow cytometer (BD Accuri C6).

### 2.10 Cell apoptosis assays

Cell apoptosis was assessed using Annexin V‐propidium iodide staining followed by flow cytometry using the Annexin V‐PI Apoptosis Detection Kit I. (Beyotime, Shanghai, China) For si‐Slc31a1 transfection and TM or CQ experiments, the cells were kept under stress conditions for 24 or 48 hours before the cell apoptosis assay. About 5 μmol/L cisplatin (HY‐17394, MCE, a chemotherapeutic drug) or 3 μmol/L TPEN (P4413, Sigma, a membrane permeable zinc chelator) treatment was used as positive controls.

### 2.11 Measurement of ROS production

Panc‐1 and MiaPaCa‐2 cells were treated with DMSO and si‐Slc31a1 mimics for 24 hours, and intracellular ROS generation was assessed by the fluorescent probe DCFH‐DA (Beyotime) using a flow cytometer in accordance with the instructions. The cells were treated with 3 mmol/L N‐Acetyl‐L‐cysteine (NAC) and si‐Slc31a1 for 12 hours.

### 2.12 Measurement of intracellular ATP production

An ATP assay kit (Beyotime) was used to measure the intracellular ATP concentration. Cells were treated with DMSO and si‐Slc31a1 mimics for 24 hours, and 1 × 10^6^ cells were trypsinized and resuspended in ultrapure water. Luciferase activity was determined after 48 hours using the Promega GLOMA 20/20 luminometer.

### 2.13 Imaging mCherry‐GFP‐LC3

Ad‐mCherry‐GFP‐LC3 adenovirus were purchased from Beyotime, mCherry‐GFP‐LC3‐transfected Panc‐1 and MiaPaCa‐2 cells were transfected with si‐Slc31a1 for 24 hours. Cells were stained with DAPI and visualized by fluorescence microscopy. GFP‐LC3‐positive and/or mCherry‐LC3‐positive cell counts and colocalization of autophagosomes and lysosomes were detected. The GFP‐LC3 quenched signal was detected in lysosomes.

### 2.14 Western blot

The cell lysate was prepared using RIPA buffer containing protease inhibitors, phosphatase inhibitors and dithiothreitol. Blots were incubated with primary antibodies containing 5% non‐fat dry milk and incubated with anti‐SLC31A1, anti‐caspase 3, anti‐LC3 or *β*‐actin followed by a secondary antibody conjugated to horseradish peroxidase. Blot images were obtained using a chemiluminescent substrate reagent and an imaging instrument (Image Quant LAS 4000 mini; GE Healthcare, Pittsburgh, PA).

### 2.15 Statistical analyses

Bars or symbols in the graph represent the means ± standard error from at least three independent experiments. Data are presented as mean ± standard deviation. The Student's *t* test (2‐tailed) was used to determine the differences between the experimental and control groups. The level of significance was set to *P* < 0.05.

## 3 RESULTS

### 3.1 Both copper levels and Slc31a1 expression correlate with the malignant degree of pancreatic cancer

**Figure 1:**
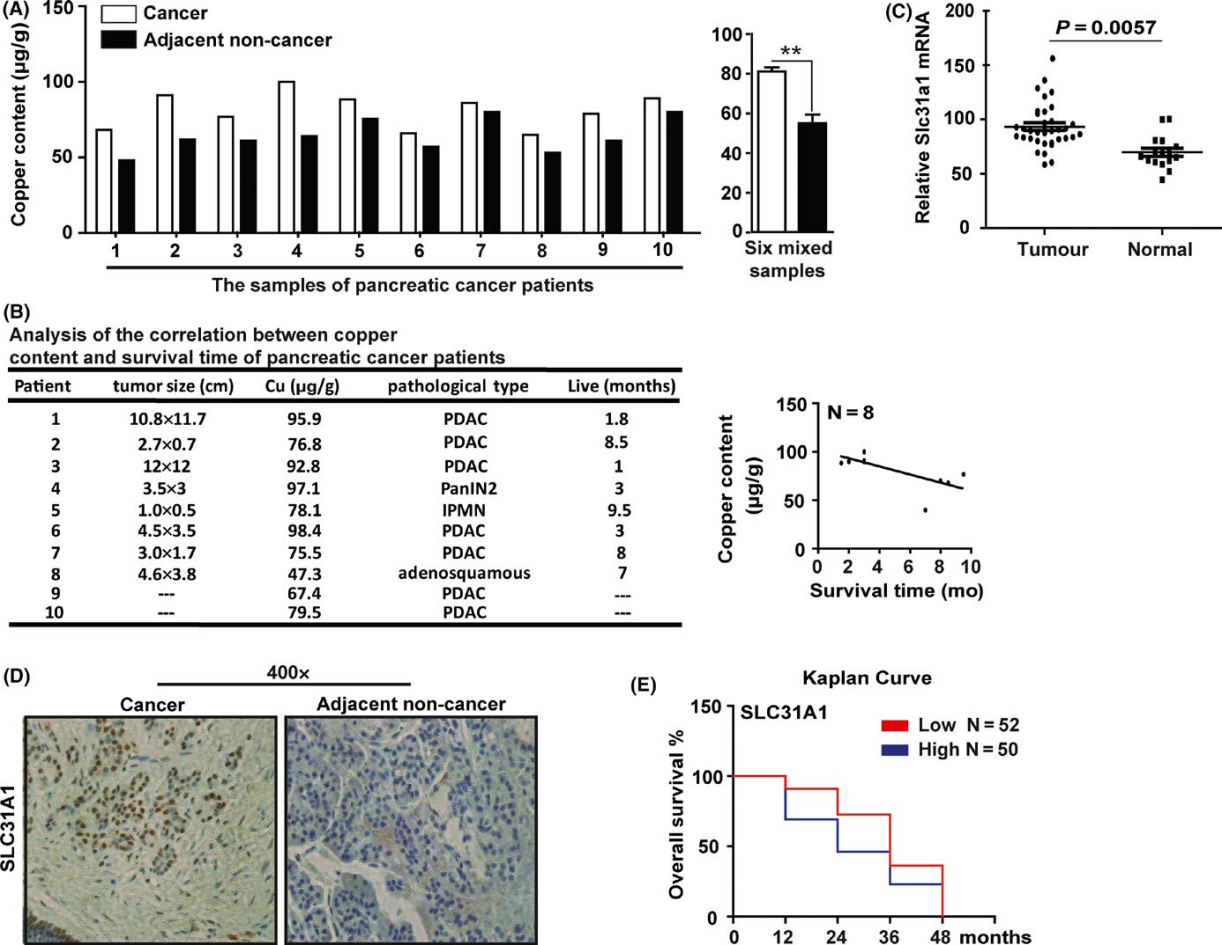
Copper content increased in pancreatic cancer tissues. A, the copper content was determined by ICP‐MS assay in pancreatic cancer (P1‐P10 and six mixed samples) and adjacent non‐cancer (AC) tissue specimens. Data shown are mean ± SD, n = 6, **P < 0.01 (Student's *t* test). B, the correlation between copper content and the survival time was analyzed in eight patients. C, GEO data analysis of Slc31a1 expression in pancreatic cancer and normal tissues. D, The SLC31A1 protein expression was examined by immunohistochemical staining. E, the correlation of Slc31a1 mRNA levels and the survival time was analyzed in 87 patients using data from the OncoLnc database

Since the content of copper in pancreatic cancer tissues is still unknown, we first detected copper levels via ICP‐MS in ten pairs of pancreatic ductal adenocarcinoma samples and their adjacent non‐cancer specimens. The results showed that copper levels were significantly higher in the cancer specimens than in those adjacent paracancer tissue specimens (Figure 1A). Further investigation of the copper content in a mixed cancer specimen and a mixed paracancer specimen from six patients (because these sample sizes were too small to be analyzed alone) showed a similar result of increased copper content in cancer tissues vs non‐cancer tissues (Figure 1A). By analyzing the correlation between copper content and the survival time in the eight independent patients, we found that the higher levels of copper in cancerous tissues corresponded with shorter survival times (Figure 1B).

It has been reported that copper is absorbed mainly by the cell surface transporter SLC31A1 in mammals, we performed quantitative RT‐PCR (qPCR) to detect the expression of Slc31a1 in pancreatic cancer and paracancer specimens. The results showed that the level of Slc31a1 mRNA expression was significantly increased in cancer tissues, and its expression was correlated to the copper level in the patient's tumour samples (Figure S1A,B). Consistent with this, the expression of Slc31a1 was found significantly higher in pancreatic cancer than in matched normal tissues based on the analysis of the NCBI database (GSE16515; Figure 1C).

Immunohistochemical staining showed that the SLC31A1 protein was more abundant in the malignant duct‐like tissues than in the normal tissues (Figure 1D). Interpretation of the transcriptome sequencing results from the MERAV database confirmed that copper transporter genes had increased expression in pancreatic cancer specimens (Figure S1C,D). Analysis of the survival curve using the data from the OncoLnc Cancer database further revealed that the higher Slc31a1 mRNA levels in the specimens correlated with lower survival times for the patients (Figure 1E). These results indicate that pancreatic cancer tissues contain a higher level of both copper content and Slc31a1 expression than the adjacent non‐cancer tissues, and their levels were associated with the degree of tumour malignancy.

### 3.2 SLC31A1‐dependent copper absorption is important for pancreatic cancer progression

**Figure 2:**
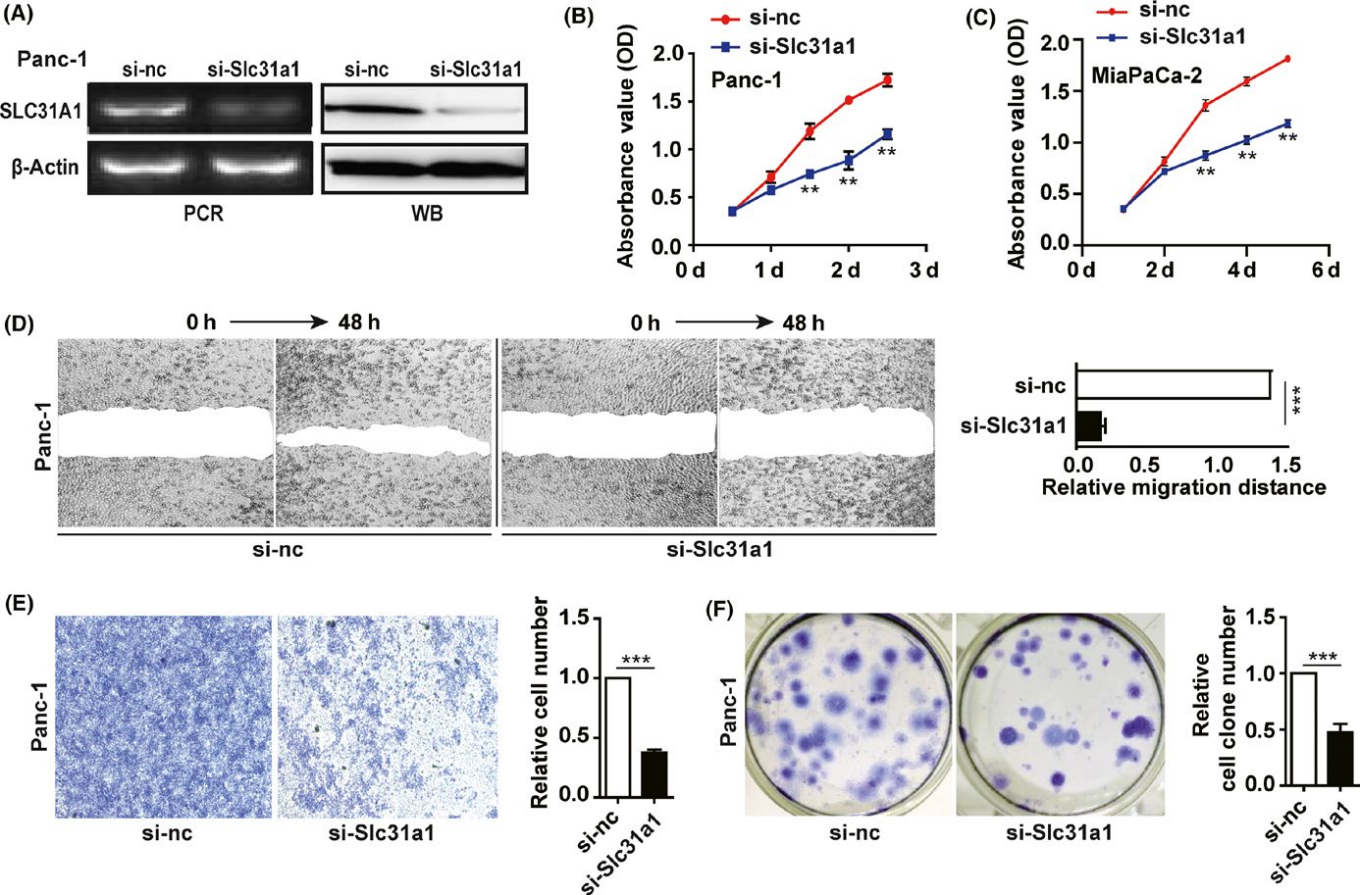
Slc31a1 knock‐down inhibited pancreatic cancer progression. A, after transfection of 50 nmol/L si‐Slc31a1 or NC for 72 h, the mRNA or protein expressions of SLC31A1 in Panc‐1 cells were measured by qPCR or Western blot, respectively. *β*‐actin was used as an internal control. B and C, the effect of si‐Slc31a1 (50 nmol/L) on Panc‐1 and MiaPaCa‐2 cell growth was measured by CCK‐8 assay for 5 days. D, the migration of Panc‐1 cells transfected with si‐nc or si‐Slc31a1 was tested via wound healing assay. The representative pictures were shown at 0 and 48 h after the wounds were made. E, the invasion of Panc‐1 cells transfected with NC or si‐Slc31a1 was studied via transwell assay. F, the colony formation capacities of Panc‐1 cells transfected with NC or si‐ Slc31a1 were determined. All results are presented as the means ± SD of values obtained in three independent experiments, n = 3, ***P < 0.001 (Student's *t* test)

Given that SLC31A1 is a major transmembrane copper transporter and its expression is increased in pancreatic cancer, we knocked down Slc31a1 in Panc‐1 cells using a previously reported siRNA^21^ (Figure 2A), and determined the intracellular copper content using ICP‐MS assay. This analysis showed that SLC31A1 interference resulted in a significant decrease in copper level in the cells, which is consistent with SLC31A1‐dependent nature of copper dysregulation in pancreatic cancer cells (Figure S2A). We next evaluated the effect of Slc31a1 knock‐down on the phenotypes of Panc‐1 and/or MiaPaCa‐2 cells. The results showed that the proliferation of pancreatic cancer cells was inhibited by si‐Slc31a1 in a time‐ and concentration‐dependent manner (Figure 2B,C and Figure S2B). When Slc31a1 knock‐down Panc‐1 cells were transfected with a full‐length SLC31A1 expression vector, cell proliferation was restored (Figure S2C). Furthermore, Slc31a1 knock‐down significantly inhibited the migration, invasion and colony formation of Panc‐1 and MiaPaCa‐2 cells (Figure 2D‐F and Figure S2D‐F). These results demonstrated that the SLC31A1‐dependent copper absorption is important for the progression of pancreatic cancer.

### 3.3 Copper deprivation renders cancer cell quiescent, but does not induce cell apoptosis

**Figure 3:**
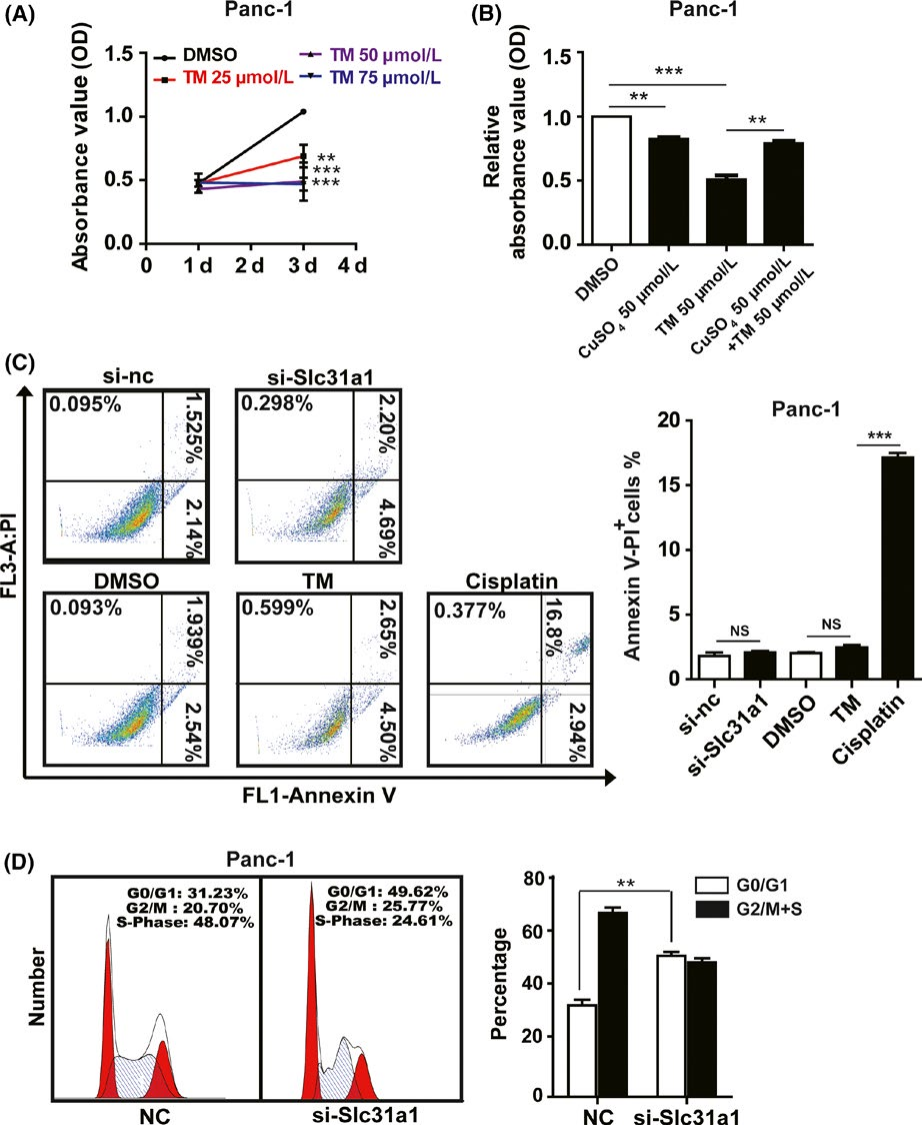
Tetrathiomolybdate (TM) inhibited pancreatic cancer progression. A, the inhibition of Panc‐1 cell proliferation by the copper chelator TM was determined by CCK‐8 assay. B, CuSO_4_ addition reversed the growth inhibition by TM. C, the effects of si‐Slc31a1 (50 nmol/L) and TM (50 μmol/L) on apoptosis in Panc‐1 cells were measured by flow cytometry, cisplatin was used as the positive control. D, the effects of si‐Slc31a1 on the cell cycle were measured by flow cytometry with PI staining. All results are presented as the means ± SD of values obtained in three independent experiments, n = 3, **P < 0.01, ***P < 0.001 (Student's *t* test)

SLC31A1‐dependent copper blockage suppressed pancreatic cancer progression, which indicated that copper deprivation could be a potential therapeutic approach. We further employed TM, a frequently used copper chelator in multiple clinical trials, to test the effect of copper deprivation on pancreatic cancer cells. The results showed that TM inhibited the proliferation of Panc‐1 cells in a dose‐dependent manner (Figure 3A), whereas 50 μmol/L copper sulphate could recover the proliferation of Panc‐1 cells treated with 50 μmol/L TM (Figure 3B). Similar to Slc31a1 knock‐down, TM treatment significantly inhibited the migration, invasion and colony formation of Panc‐1 and MiaPaCa‐2 cells (Figure S3A‐C).

We further determined cell apoptosis after copper deprivation in Panc‐1 and MiaPaCa‐2 cells induced by TM or si‐Slc31a1. Flow cytometry analysis demonstrated that both TM and si‐Slc31a1 did not promote cell apoptosis (Figure 3C and Figure S3D), which was confirmed by Western blot analysis of active caspase‐3 (Figure S3E). Moreover, cell cycle analysis indicated that TM or si‐Slc31a1 increased the G0/G1 phase of pancreatic cancer cells (Figure 3D). These results indicated that copper deprivation suppressed the proliferation of pancreatic cancer cells and did not promote cell apoptosis, which allowed them to enter a dormant state.

### 3.4 TM remove could recover the malignant characteristics of pancreatic cancer cells

**Figure 4:**
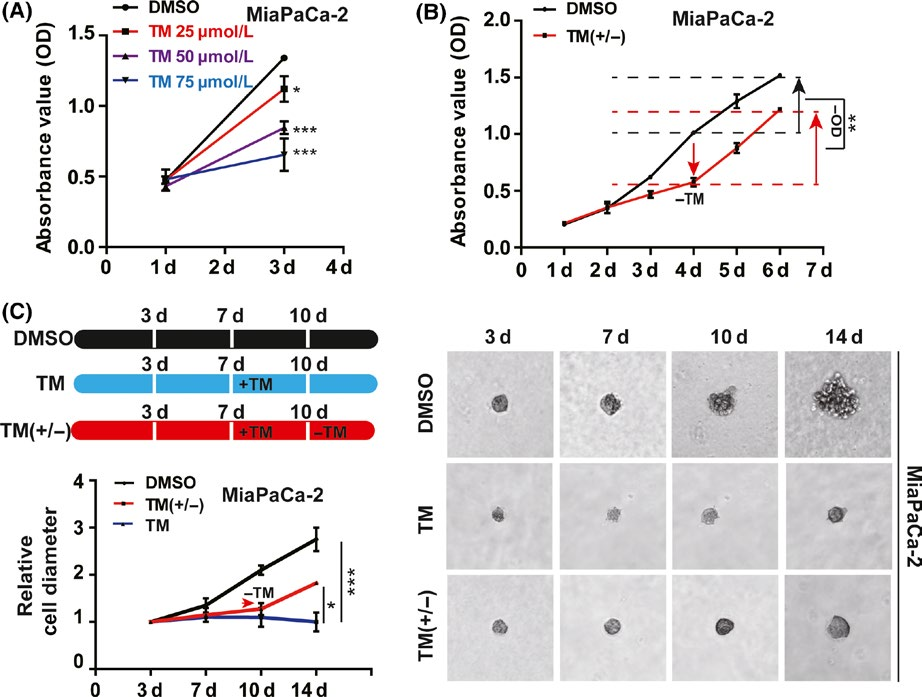
The removal of tetrathiomolybdate (TM) restored the proliferation of pancreatic cancer cells. A, the inhibition of the growth of MiaPaCa‐2 cells by TM (50 μmol/L) was determined by CCK‐8 assay. B, Cell proliferation was tested after TM was withdrawn for 24 or 48 h. DMSO‐treated cells served as the negative control. C, the growth of MiaPaCa‐2 cell spheres was inhibited by TM and recovered after TM withdrawal. A total of 50 μmol/L TM was added on the seventh day after the cells were plated and then removed on the tenth day. All results are presented as the means ± SD of values obtained in three independent experiments, n = 3, *P < 0.05, **P < 0.01, ***P < 0.001 (Student's *t* test)

Since TM or si‐Slc31a1 did not kill the pancreatic cancer cells but converted them into a dormant state, we further explored the effect after TM withdrawal. When TM was removed from the culture media for 48 hours, the proliferation of MiaPaCa‐2 was not only recovered but also became more rapid (Figure 4A,B). We then adopted a sphere‐forming assay to evaluate the effect of the addition or withdrawal of TM on MiaPaCa‐2 malignant characteristics. The results showed that TM treatment for 7 days did not lead to cell death, and cell proliferation was recovered and regained rapid growth upon TM removal (Figure 4C).

### 3.5 Copper deprivation leads to cell cycle arrest increased ROS and decreased ATP production

To explore the potential mechanism by which the pancreatic cancer cells entered a silent state under copper deprivation, we first searched the GEO dataSets and found the microarray data regarding TM‐treated breast cancer cells. We analyzed the altered genes via KEGG pathway and found that the first altered 250 genes are concentrated in the physiological processes of cellular metabolism (Figure S4A). We classified these metabolic genes and found that most of them were involved in cellular oxidative respiration and energy production, and these genes were downregulated after TM treatment (Figure S4B,C). We hypothesized that the cell cycle arrest after copper absorption blockage might be caused by the increase in oxidative stress and mitochondrial dysfunction.

**Figure 5:**
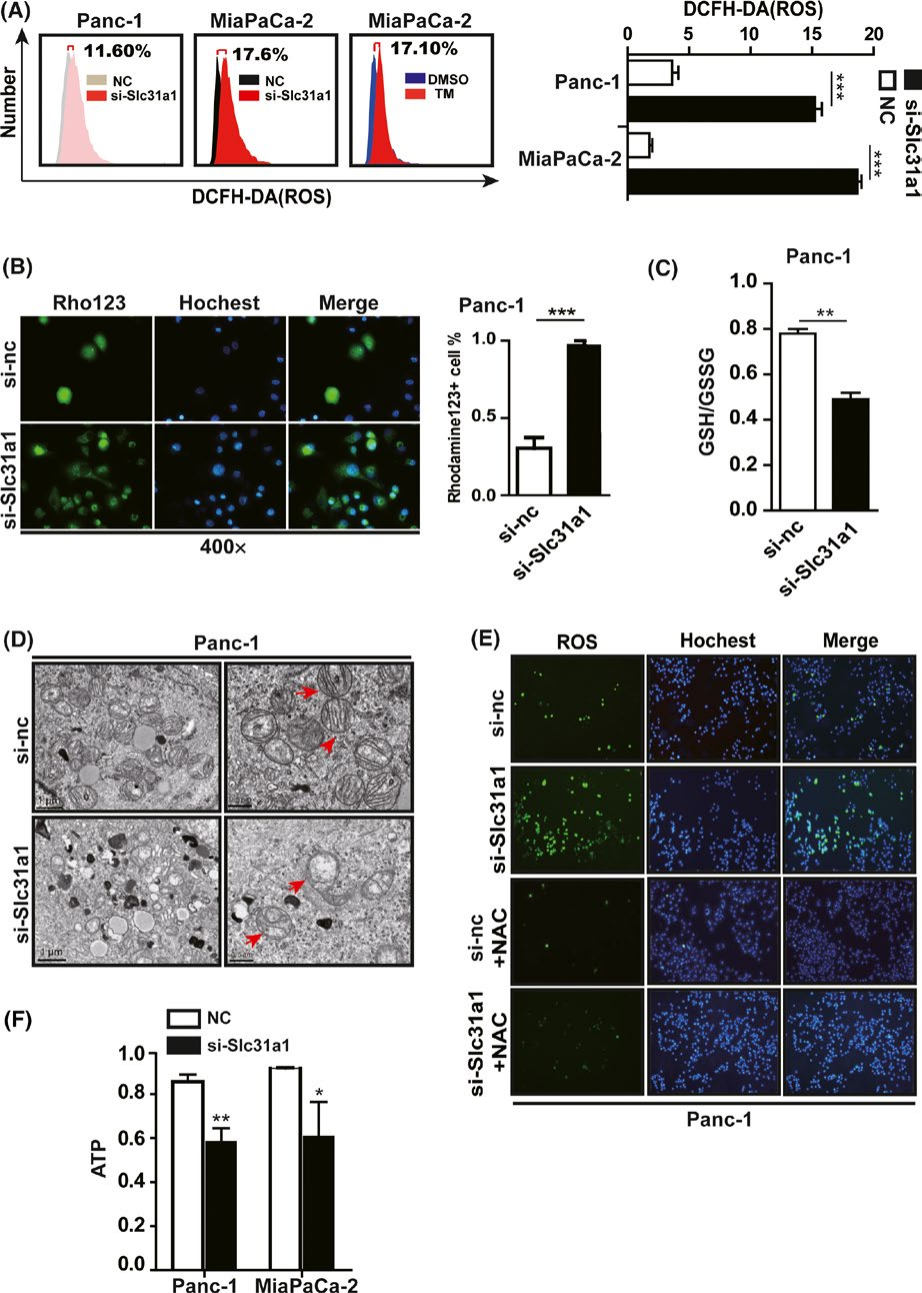
Copper deprivation by si‐Slc31a1 and tetrathiomolybdate (TM) inhibited the mitochondrial activity of pancreatic cancer cells. A, The ROS levels of Panc‐1 and MiaPaCa‐2 cells that were transfected with 50 nmol/L si‐Slc31a1 or treated with 50 μmol/L TM for 24 h were measured by flow cytometry. B, Mitochondrial membrane potential of Panc‐1 transfected with NC or si‐Slc31a1 (50 nmol/L) was tested. Rhodamine123 was used to test mitochondrial membrane potential. C, The GSH/GSSG ratio of Panc‐1 cells that was induced by Slc31a1 interference was presented via GSH/GSSG kit instructions. D, the mitochondrial morphology of Slc31a1 knocked down Panc‐1 cells was presented via transmission electron microscope. The red arrow showed mitochondrial morphology. E, The Slc31a1 knocked down Panc‐1 cells were treated with NAC (1 mmol/L) for 24 h, and ROS levels were then measured via fluorescence detection. F, The ATP levels in Panc‐1 and MiaPaCa‐2 cells were shown after the cells were transfected with si‐Slc31a1 or NC for 48 h. All results are presented as the means ± SD of values obtained in three independent experiments, n = 3, *P < 0.05, **P < 0.01, ***P < 0.001 (Student's *t* test)

Using the DCFH‐DA probe and flow cytometry analysis, we found that si‐Slc31a1 or TM was capable of significantly increasing cellular ROS levels at 48 hours after the treatment of Panc‐1 and MiaPaCa‐2 cells (Figure 5A and Figure S4D). As shown in (Figure 5B), transfection of si‐Slc31a1 increased the intracellular Rhodamine123 staining in Panc‐1 cells accompanied by ROS increase, indicating a decrease in mitochondrial permeability and a decrease in glutathione (GSH). An increase in oxidized glutathione (GSSG) was also found in si‐Slc31a1 transfected Panc‐1 cells (Figure 5C). Accordingly, we observed the change in mitochondrial morphology induced by si‐Slc31a1 on the basis of electron microscopy (Figure 5D).

Furthermore, when the increased cellular ROS was ameliorated with N‐acetyl‐L‐cysteine (NAC), the cell proliferation was recovered (Figure 5E). To establish if inhibition of copper absorption resulted in additional metabolic dysfunctions in pancreatic cancer cells, we examined the bioenergetics properties of si‐Slc31a1‐transfected or TM‐treated Panc‐1 cells. The results showed that the cellular ATP levels were noticeably reduced (Figure 5F).

### 3.6 Impaired copper absorption increases autophagy in pancreatic cancer cells

**Figure 6:**
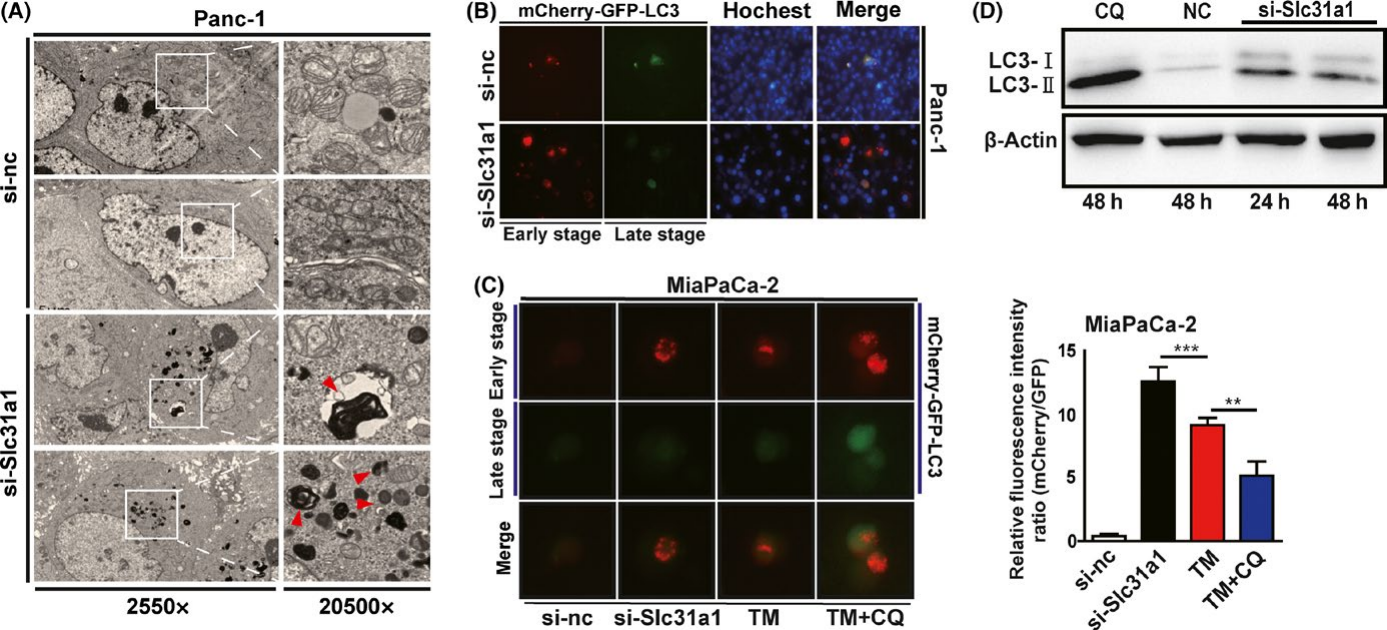
Copper deprivation increased pancreatic cancer cell autophagy. A, the autophagy of Panc‐1 cells that was induced by Slc31a1 interference was presented via transmission electron microscope. B and C, Fluorescence detection of mCherry‐GFP‐LC3 in TM‐treated or Slc31a1 knocked down Panc‐1 and MiaPaCa‐2 cells. Red is representative of an early stage of autophagy, and green quenching indicates the increased function of lysosomes. Data shown are mean ± SD, n = 3, **P < 0.01, ***P < 0.001 (Student's *t* test). D, Western blot detected LC3I and LC3II in si‐Slc31a1‐transfected Panc‐1 cells

It has been demonstrated that intracellular metabolic dysfunction might lead to cell apoptosis or autophagy. Because copper deprivation caused a small increase in cell apoptosis, we further tested cell autophagy after blocking copper absorption. Electronic microscopy results showed that a large amount of autophagosomes was found in si‐Slc31a1‐transfected Panc‐1 cells (Figure 6A). The subcellular location of LC3, mainly in the autophagosomes, was detected by using a fluorescence microscope after transfecting a reporter vector mCherry‐GFP‐LC3 in both TM and Ctr‐1 knock‐down Panc‐1 or MiaPaCa‐2 cells (Figure 6B,C). Immunoblot analysis showed that the ratio of LC3II/I was significantly increased (Figure 6D). All these data indicated that the copper inhibition induced by TM or si‐Slc31a1 resulted in a significant increase in cellular autophagy in pancreatic cancer cells.

### 3.7 Increased autophagy induced by copper deprivation suppresses pancreatic cancer cell death

**Figure 7:**
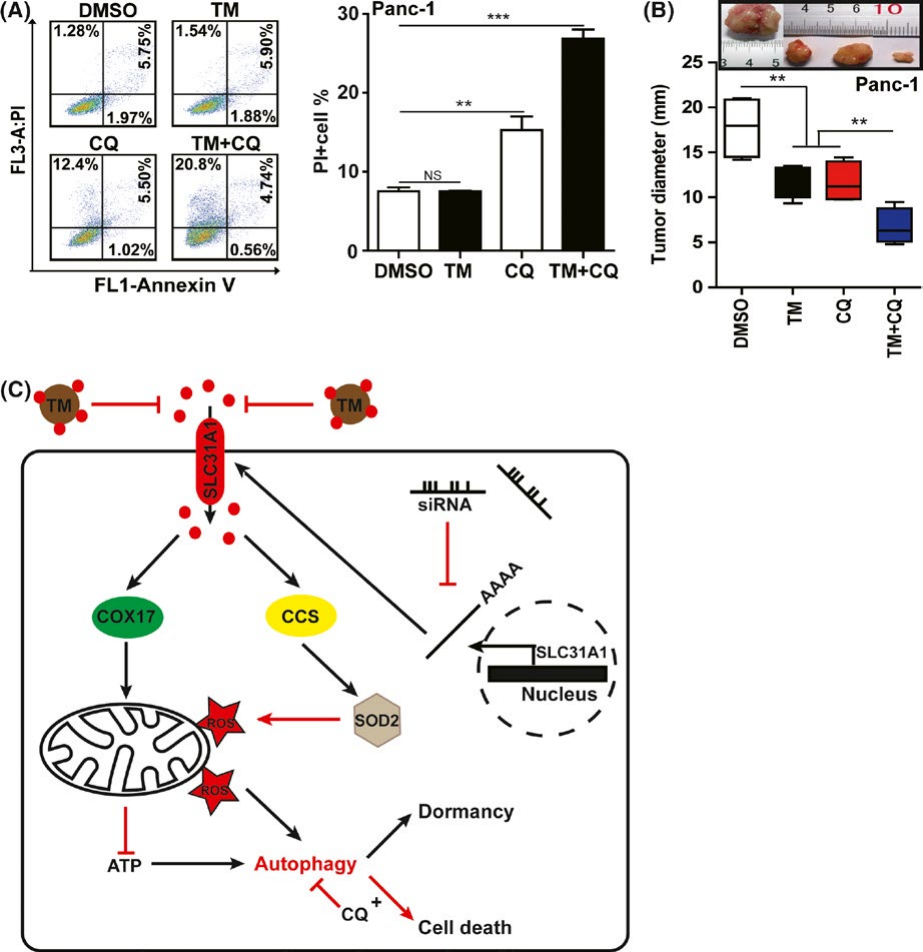
A combination of copper deprivation with autophagy inhibition repressed pancreatic cancer progression. A, Cell apoptosis treated with tetrathiomolybdate (TM) (50 μmol/L) combined with CQ (10 μmol/L) was measured by flow cytometry in Panc‐1 cells. Data shown are mean ± SD, n = 3, **P < 0.01, ***P < 0.001 (Student's *t* test). B, the effects of TM or CQ individually or TM combined with CQ treatment on tumour formation were tested by transplanting treated Panc‐1 cells into NPG immune‐deficient mice. For TM and CQ treatments, the NPG mice were injected subcutaneously with 2 × 10^6^ Panc‐1 cells, and the xenografted mice were then administered TM, CQ, or a combination of TM and CQ in their water. Twenty days after transplantation, the tumours were removed and weighed. Each group included three mice. The xenografted tumours shown here were from one of three groups. The sizes of the tumours in each group were calculated and compared using Student's *t* test, n = 3, **P < 0.01, ***P < 0.001 (Student's *t* test). C, Schematic diagram of pancreatic cancer cells resisting the effect of copper shortage caused by SLC31A1 interference or TM treatment through entering a dormant state and increasing autophagy

To elucidate the role of increased autophagy induced by copper deprivation in pancreatic cancer cells, we employed the autophagy activator rapamycin (Rapa) and the autophagy inhibitor CQ in our subsequent experiments (Figure S5A). In contrast, the combination of TM with the autophagy inhibitor CQ led to a significant increase in cell death in Panc‐1 cells, indicating that cell necrosis was a major way of death (Figure 7A). Furthermore, CQ treatment alone or TM and CQ combined treatment increased intracellular ROS levels and decreased ATP levels (Figure S5B,C). These results indicated that increased autophagy contributes to the resistance of cell death, which could be caused by copper‐deprived mitochondrial dysfunction.

We further tested the effect of copper deprivation induced by TM and/or autophagy inhibition induced by CQ in tumour xenograft. The results showed that CQ or TM treatment alone could inhibit cancer growth to certain extent; however, combined treatment could more effectively decrease the tumour size than alone treatment (Figure 7B and Figure S5D). Concurrently, we determined the weight and the AST/ALT ratio of the xenografted mice administered CQ and TM and did not find significant changes in these detected indexes in any of the treatment groups (Figure S5E,F). These results indicated that the combination of autophagy inhibition and copper deprivation was a potential strategy for the therapy of pancreatic cancer.

## 4 DISCUSSION

In this study, we found that SLC31A1‐dependent copper absorption was elevated in pancreatic cancer samples. Additionally, the levels of copper and SLC31A1 were negatively correlated with patient survival. Copper suppression via SLC31A1 knock‐down or TM treatment repressed pancreatic cancer cell proliferation, migration, and invasion, which demonstrated that elevated copper plays an essential role in pancreatic cancer progression. The effect of copper on pancreatic cancer can be shown in Figure 7C. However, copper deprivation from pancreatic cancer cells affected their mitochondrial function, decreasing ATP and increasing the ROS, which caused the increase in cell autophagy. The fact that autophagy inhibitors CQ effectively increased the death rate of cancer cells suggest that autophagy contributes to the resistance to cell death.

Increasing evidence demonstrates that elevated copper is essential for the growth and metastasis of solid tumours.^6^, ^7^, ^8^, ^9^ In addition to being a ligand of SOD1 and COX4, copper is found to be essential for the activation of MEK‐ and BRAF‐driven tumour growth.^12^, ^22^ Furthermore, copper is required for the activities of Memo and lysyl‐oxidase (LOX), which have been demonstrated to be involved in cancer cell migration and invasion.^23^, ^24^, ^25^, ^26^ However, the mechanisms by which copper elevation in cancer cells remain unclear. In this study, we revealed that the increased expression of SLC31A1 was responsible for the increase in copper in pancreatic cancer cells. SLC31A1 has been paid close attention on cisplatin uptake in the past years,^27^, ^28^, ^29^ whereas its role in cancer development has not been fully understood. Recently, it has been reported that the expression of SLC31A1 increases in several tumours, including prostate, colorectal, breast cancer and melanoma.^10^, ^11^, ^30^, ^31^ Lowering the expression of SLC31A1 could inhibit the proliferation of cancer cells,^9^, ^10^, ^12^ which is consistent with our results. Our data and bioinformatic analysis further revealed that the levels of Slc31a1 mRNA were negatively correlated with the survival time of pancreatic cancer patients. These results indicated that SLC31A1 is a noteworthy gene for cancer diagnosis and therapy.

Because of its specific role in cancer development, copper has become a promising target for the development of anti‐cancer drugs. Recent phase II clinical trials showed that the copper chelator TM was beneficial for the prevention relapse in patients with triple‐negative breast cancer.^32^, ^33^ Nevertheless, several other trials indicated that copper deprivation via TM or other chelators could not benefit patient's survival in several advanced cancers.^34^, ^35^ Our results demonstrate that copper suppression is not sufficient to kill pancreatic cancer cells, but rather triggers the cancer cells to enter a dormant state, which is supposed to be in favour of prevention of recurrence in TM‐treated breast cancer cells.^32^, ^33^ However, dormancy is also a strategy for cancer cells to resist the adverse situation.^36^ In accordance with this assumption, we discovered that once TM was withdrawn, the cancer cells resumed growth at an even faster rate. These findings could explain why copper chelators are not ideal for the clinical trials of advanced cancers.

Here, we first discovered that blocking the copper absorption resulted in increased cell autophagy, which has been proven to be not only a pathway for getting nutrients to cancer cells but also a stress response strategy.^21^, ^37^ In this study, we further demonstrated that copper depletion increased autophagy and played a stress‐resistant role in avoiding cell death after copper deprivation. Accordingly, autophagy inhibition abolishes the protection of autophagy against the stress from copper deficiency. As shown in our results, the death of pancreatic cancer cells was significantly increased by simultaneous treatment with the autophagy inhibitors CQ and TM.

## CONFLICT OF INTEREST

All co‐authors implicated in this research approved this article to be published. The authors declare that they have no conflict of interest.

## AUTHOR CONTRIBUTIONS

Ze Yu performed experiments and figure drawing. Rongtao Zhou and Yicheng Zhao performed part of experiments and data analysis. Hao Liang and Tai Sheng collected and analyzed clinical specimens. Yi Pan performed part of bioinformatic analysis. Jin‐san Zhang revised the manuscript. Chun‐Bo Teng and Liang Jin conceived and designed the project and wrote the paper.
