# Supplementary material for: Blockage of SLC31A1‐dependent copper absorption increases pancreatic cancer cell autophagy to resist cell death
Source: Cell Prolif. 2019 Jan 31;52(2):e12568. doi: 10.1111/cpr.12568 (PMC6496122; doi:10.1111/cpr.12568)
Supplement: Supplementary file 1 [file CPR-52-e12568-s001.doc]

**Blockage of SLC31A1-dependent Copper Absorption Increases Pancreatic Cancer Cell Autophagy to Resist Cell Death**


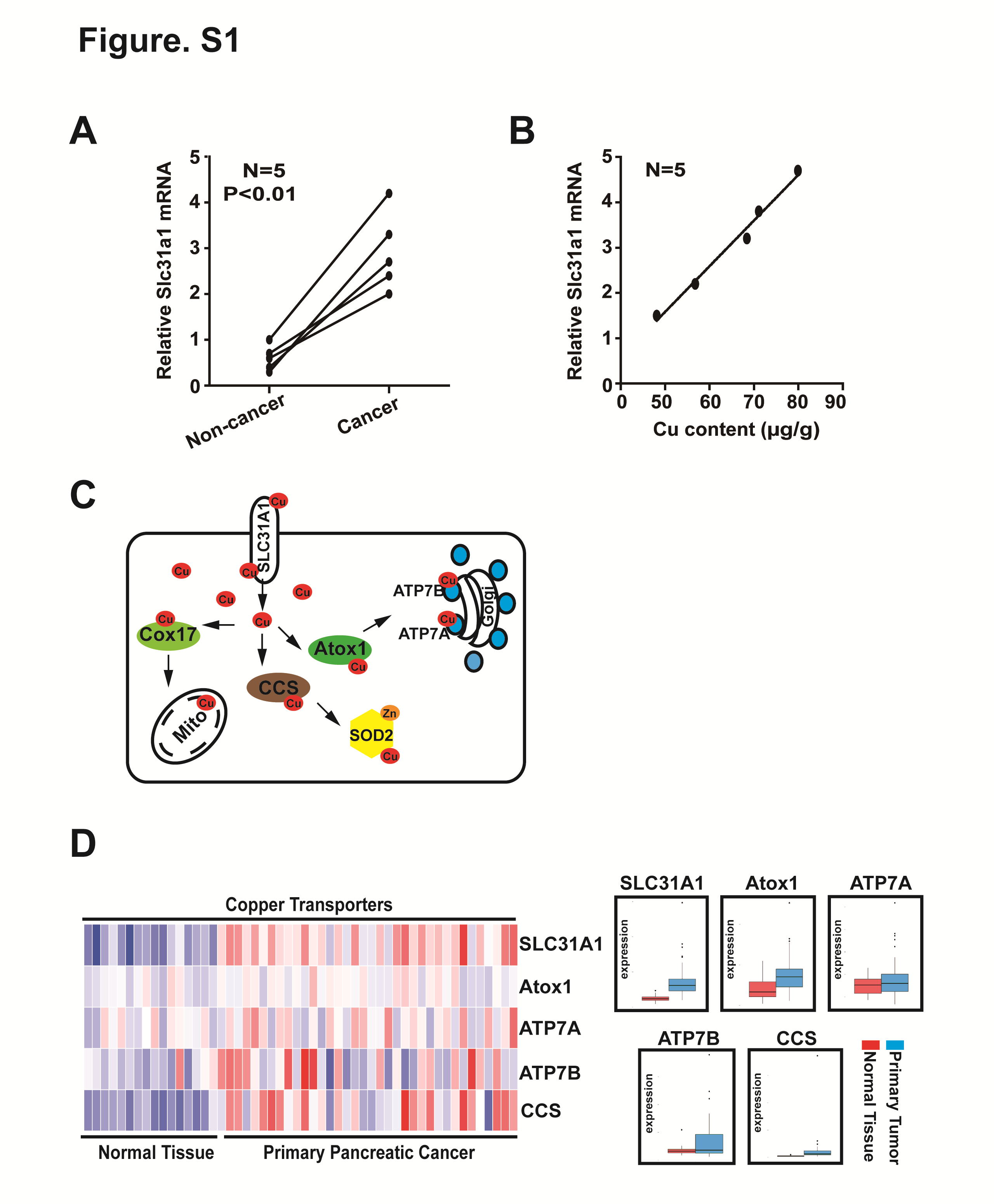


**FIGURE S1. Copper transporters were up-regulated in pancreatic cancer samples.** (A&B) The correlation between Cu content and Ctr1 mRNA expression in pancreatic cancer and their corresponding paracancer tissue samples were shown. (C) The diagram of copper chaperones in cells. (D) The Heatmap and Box maps showed the expression levels of the copper transporter genes (Ctr1, Atox1, ATP7A, ATP7B and CCS) in normal and carcinoma pancreatic tissues by analyzing data from the MERVE database.


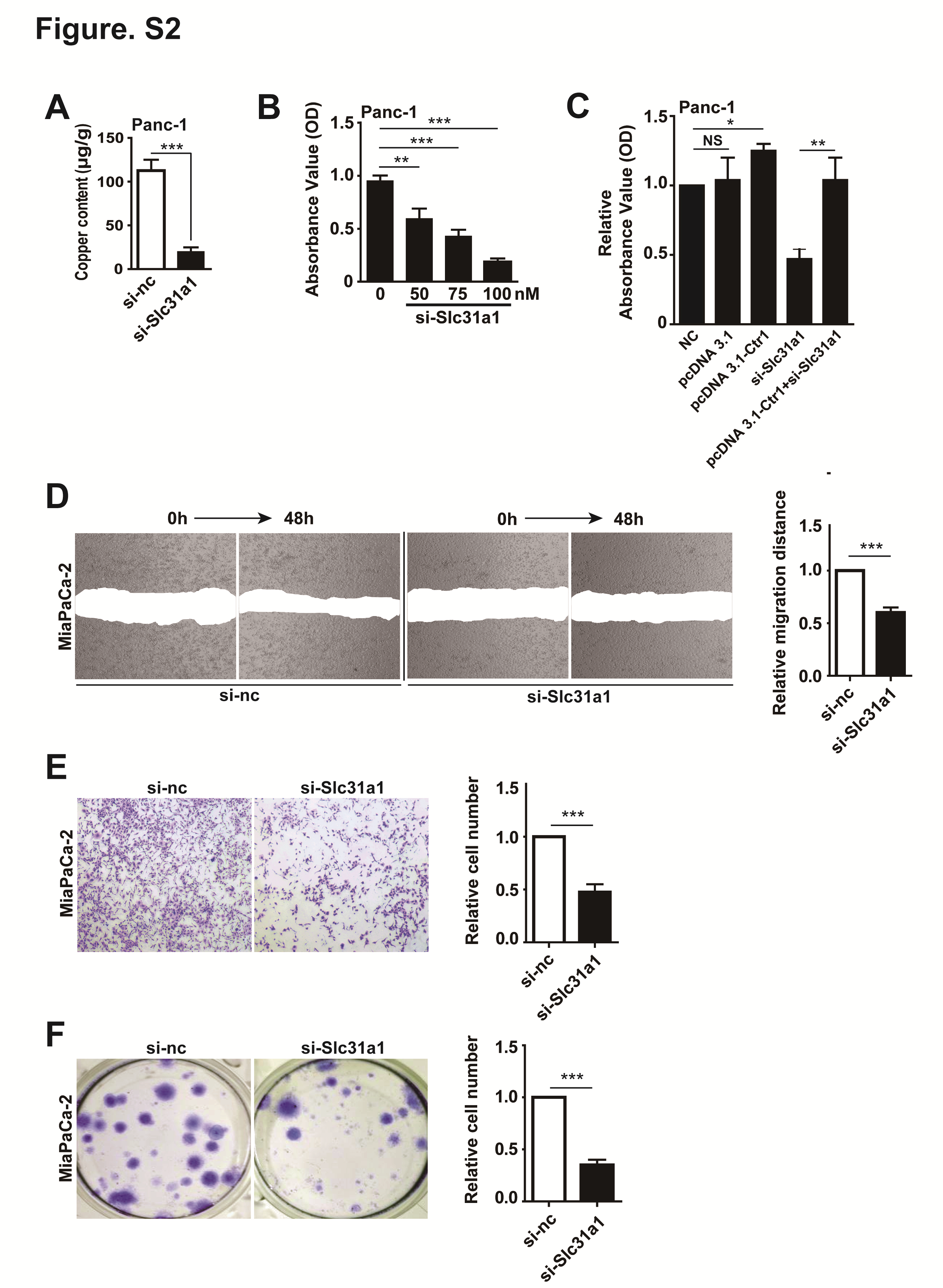


**FIGURE S2. Effect of Ctr1 interference on pancreatic cancer development.** (A) The copper content was determined by ICP-MS assay after transfecting with si-Ctr1 (50nM). (B) Panc-1 cells transfected with different concentrations of si-Ctr1 (0～100nM) were measured by CCK-8 assay. (C) The transfection of pcDNA3 1-Ctr1 into Ctr1 knock-down Panc-1 cells reversed their growth inhibition. (D) The migration of MiaPaCa-2 cells was tested via the wound healing assay after Ctr1 was knocked down. The representative pictures were shown at 0 h and 48h after the wounds were made. (E) The invasion of MiaPaCa-2 cells transfected with NC or si-Ctr1 (50nM) was determined via transwell assay. (F) Plate colony formation of MiaPaCa-2 transfected with NC or si-Ctr1 (50nM) was tested. All results were presented as the mean ± S.D., the symbols * and ** and *** represent statistical significance at p < 0.05, p < 0.01, and p < 0.001, respectively.


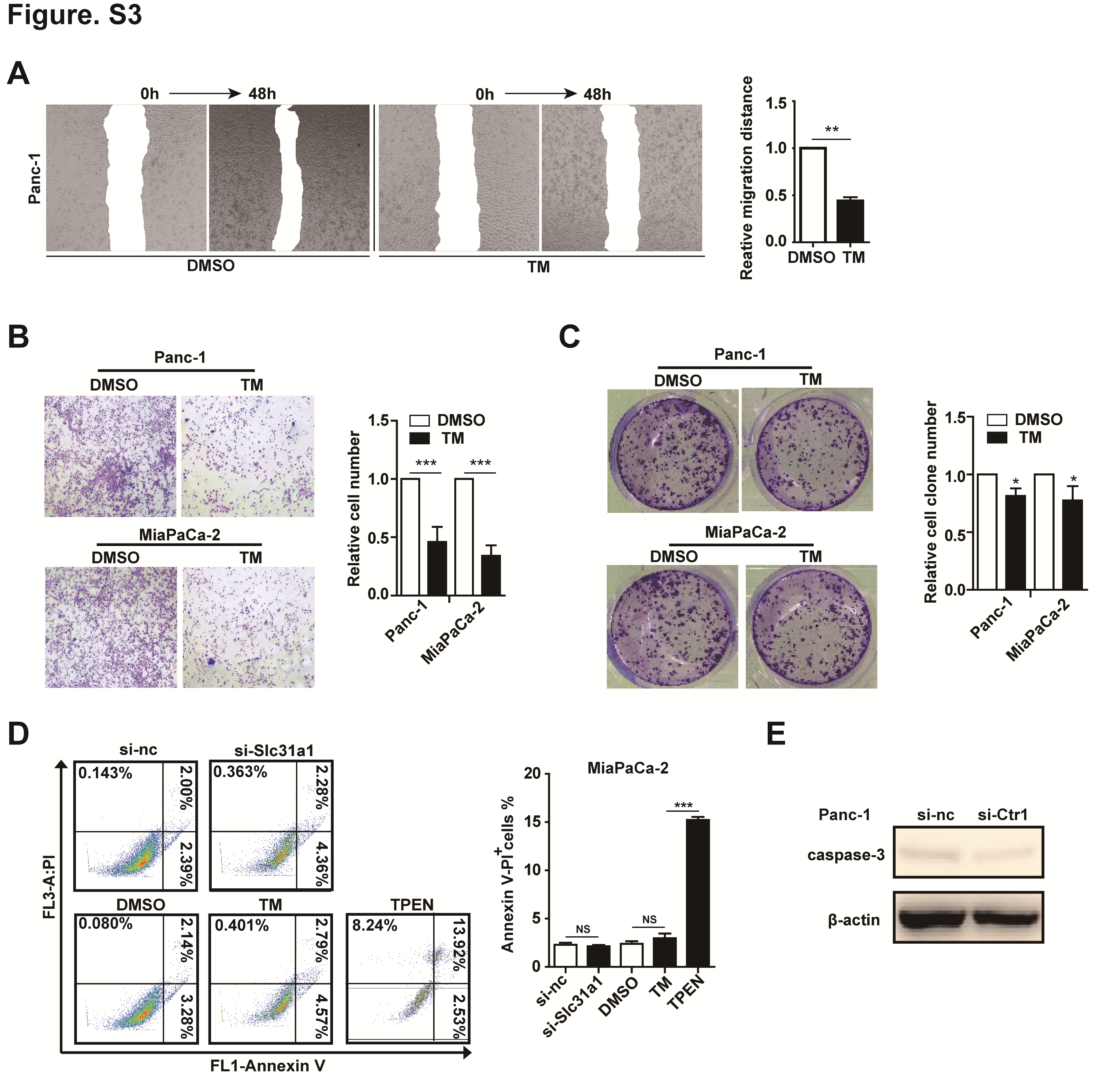


**FIGURE S3. Effect of TM on pancreatic cancer progression.** (A) The migration of Panc-1 cells treated with 50 µM TM for 48 h. The representative pictures were shown at 0 h and 48 h after the wound was made. (B) The invasion of Panc-1 and MiaPaCa-2cells treated with 50 µM TM for 48 h. (C) Plate colony formation of Panc-1 and MiaPaCa-2cells treated with 50 µM TM for 48 h. (D) The effects of si-Slc31a1 (50 nM) and TM (50 µM) on cell apoptosis in MiaPaCa-2 cells were measured by flow cytometry, TEPN was used as the positive control. (E) The effects of si-Ctr1 (50 nM) on apoptosis in Panc-1 cells were measured by Western blot detection of caspase-3. All results were presented as the means ± S.D. of values obtained in three independent experiments, n =3, *P<0.05, **P<0.01, ***P<0.001 (Student’ s t-test).


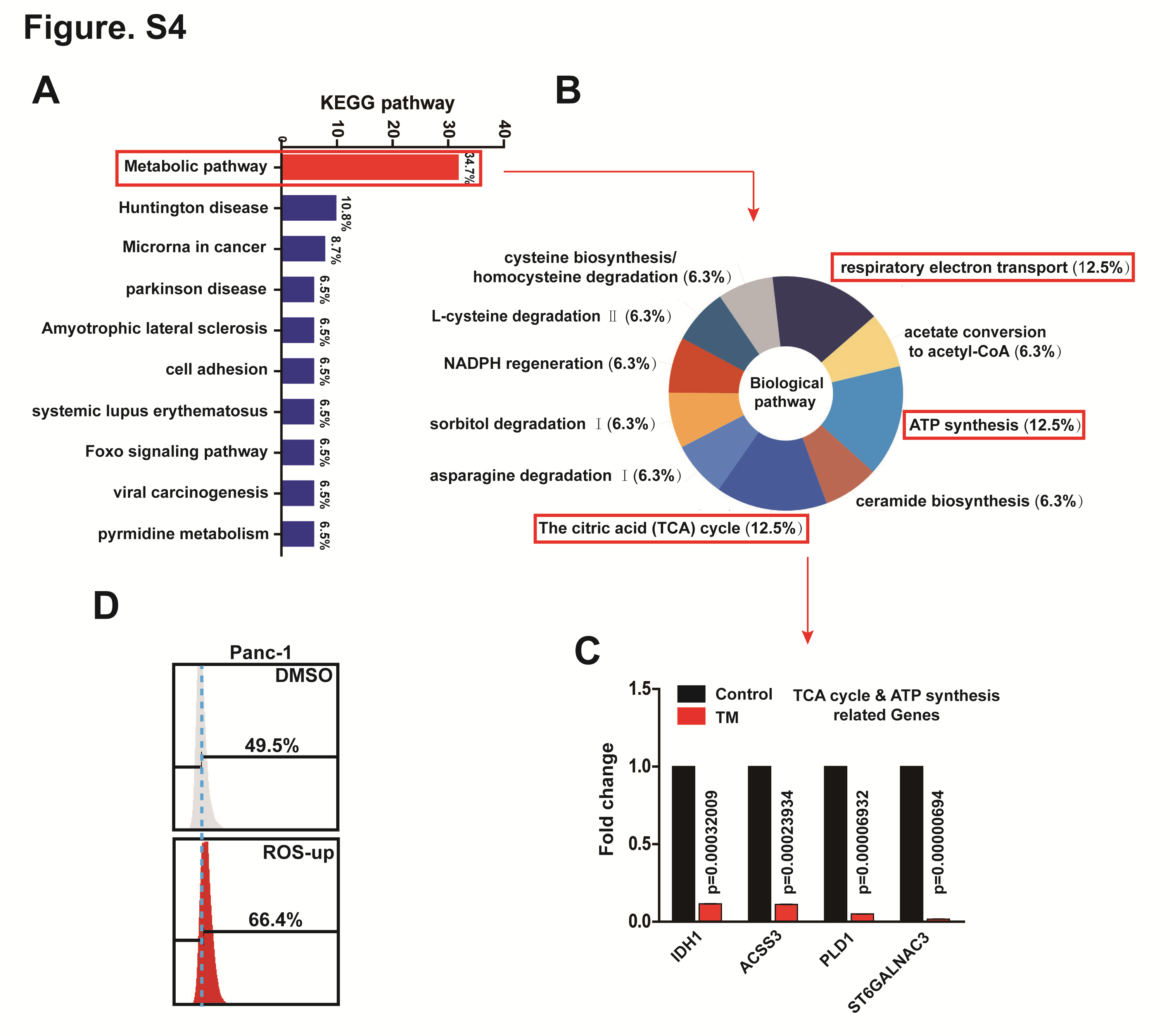


**FIGURE S4. Analysis of TM on gene expression of breast cancer cells using GEO DataSets.** (A) The top 10 pathways enriched from EGO microarray data (GSE77515). (B) Biological processes involved in highly differential genes were analyzed according to Fig. A. (C) Differential expression of energy metabolism related genes in the control and TM-treated group (GSE77515). (D) The ROS levels of Panc-1 cells transfected with 5 µM ROS-up (positive control) for 6h were measured by flow cytometry.


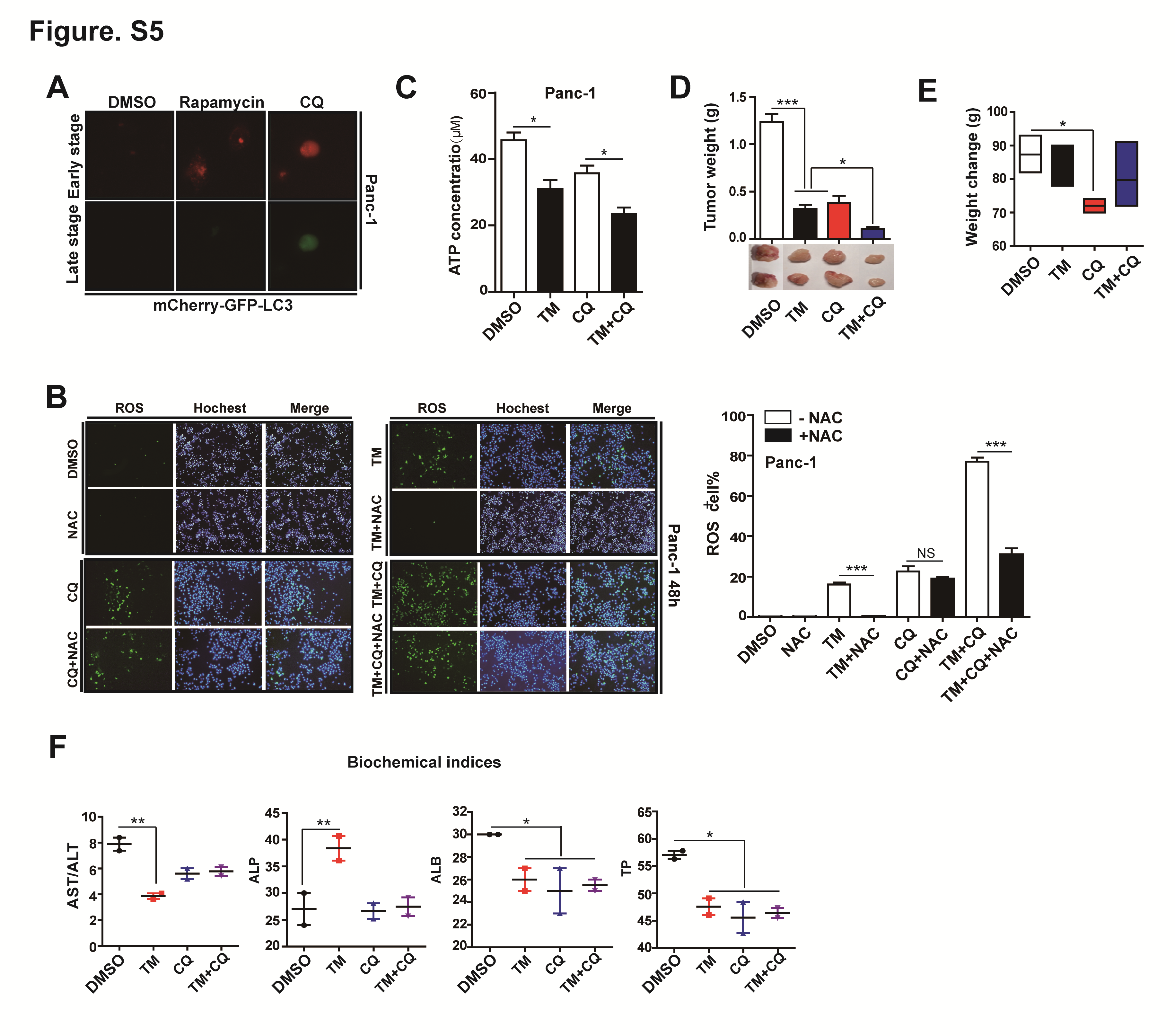


**FIGURE S5. Effect of copper deprivation combined with autophagy inhibitor on pancreatic cancer progression.** (A) Autophagy of Panc-1 cells was determined by fluorescence detection of mCherry-LC3-GFP after Rapamycin or CQ treatment. Red is representative of an early stage of autophagy, and green quenching indicates the increased function of lysosomes. (B) The ROS levels of Panc-1 cells treated with NC, TM, CQ, or NAC alone, TM and NAC, CQ and NAC, TM and CQ, or the combination of TM, CQ and NAC for 48 h. (C) The ATP levels of Panc-1 cells in TM, CQ, or the combination of TM and CQ treatment groups. (D) The effects of TM or CQ individually or TM combined with CQ treatment on tumor formation were tested by transplanting treated Panc-1 cells into NPG immune-deficient mice. The weight of tumors in each group were calculated and compared using Student’s t-test. (E) Body weight changes in 6 groups of mice were shown. (F) Liver function indexes (AST/ALT, TP, ALP, ALB) in 4 groups of mice were detected. The results were presented as the mean ± S.D. of values obtained in three independent experiments. n =3, *P<0.05, **P<0.01, ***P<0.001 (Student’ s t-test).
